# Conformity of package inserts information to regulatory requirements among selected branded and generic medicinal products circulating on the East African market

**DOI:** 10.1371/journal.pone.0197490

**Published:** 2018-05-22

**Authors:** Hiiti B. Sillo, Nelson E. Masota, Sunday Kisoma, Lembit Rago, Veronica Mgoyela, Eliangiringa A. Kaale

**Affiliations:** 1 Tanzania Food and Drugs Regulatory Authority, Dar Es Salaam, Tanzania; 2 Department of Medicinal Chemistry, School of Pharmacy, MUHAS Dar Es Salaam, Tanzania; 3 Pharmaceutical R&D Laboratory, School of Pharmacy, MUHAS Dar Es Salaam, Tanzania; 4 Council for International Organizations of Medical Sciences (CIOMS), Geneva, Switzerland; Universite de Bretagne Occidentale, FRANCE

## Abstract

**Background:**

Availability of correct and adequate information about medicines is an important aspect in ensuring rational use of medicines and hence facilitating safety and expected efficacy of medicines during therapy. Package inserts have proven to be a good source of information to the prescribers and patients whereby they have been useful in highlighting important information pertaining proper use and handling of the medicines. The present study was aimed at establishing the extent to which package inserts of medicines circulating on the markets of the East African Community (EAC) Partner States conform to medicines information requirements as established in the harmonized guidelines as well as national guidelines.

**Methods:**

A total of 99 package inserts from six (6) types of medicines namely Albendazole, Artemether/Lumefantrine (ALu), Ciprofloxacin, Paracetamol, Amoxicillin and Metronidazole were purposefully collected from three EAC Partner States: Kenya, Tanzania and Uganda. The medicines were selected based on their indications as first line treatments, high rates of utilization within the medicines supply system and their positions in treatment of diseases of public importance across EAC Partner States. The inserts were evaluated on the availability of information regarding fifteen (15) parameters as extracted from the EAC harmonized guidelines for registration of medicines. Moreover, comparisons were made between the percentage conformity of the branded versus generic products, markets from which the samples were collected, origin of the manufacturer and type of medicine.

**Results:**

Majority (93.9–100%) of the medicines’ package inserts highly conformed to the inclusion of the information regarding the description and composition of the medications, indications, dosage and methods of administration, warnings and precautions, contraindications and storage conditions. However, the information on handling and disposal, container package description, excipients used, clinical pharmacology of the medicines, and directions regarding overdose ranked the least in conformance with conformity ranging from 13.1–52.5%.

The parameter with the lowest observed percentage conformity among the branded products scored 50% as compared to 10.8% among the generic products. Moreover, there was no significant difference (P<0.05) in the percentage conformity of the package inserts collected from each of the three Partner States as compared to the average from studied medicines. A generally good conformity was observed among medicines manufactured by European based manufacturers as compared to those based in Asia and EAC Partner States. In addition, PIs of Albendazole, Ciprofloxacin, Amoxicillin and Artemether/Lumefantrine did show overall high conformity across most of the product information requirements.

**Conclusion:**

Our study revealed the existence of a significant number of medicinal products circulating on the markets of EAC Partner States without necessary compliance with all product information requirements. We therefore recommend that NMRAs ensure thorough pre-market assessment of product information as well as strengthening their post marketing surveillance to ensure that medicines circulating on the market comply to medicines information requirements at all times. Emphasis should also be given to manufacturers on the importance of inclusion of appropriate and adequate product information for the safety of patients, including advocating for inclusion of patient-friendly and easy to understand medicines information.

## Introduction

Ensuring that scientifically justified and proper information is approved and communicated to the prescribers, dispensers and patients as a result of medicines registration process is a critical aspect of medicines regulation. Medicine is "product plus information"; hence presence of adequate and correct information in the package inserts supplied in commercial packs of the medicinal products is essential [1, 2] The information provided in the PIs is much needed to allow the patients to better understand their medicines resulting in higher compliance and decreased chances of negative effects related to medicines use. The limited contact duration between the patients and health care providers, makes the availability of written information an immediate and readily available means of getting all the important information regarding the medicine(s) at hand [3].

Prescribers and dispensers have been cited by the patients as the preferred sources of information about medicines; however the need to complement the information with package inserts (PIs) has as well been identified [4]. Provision of package inserts has been highly associated by increase in knowledge about the medications by both health professionals and patients. However, more attention need to be given to non-prescription medicines, where there is often no other reliable source of information [5–7]. Users of medicines have identified PIs as helpful sources of information and they indicated the need for more detailed PIs with simplified information as they are often faced with difficulty in understanding and remembering the included information [8–10]. Other studies have indicated information of side-effects of medicines to be the most desired information by patients/clients [11]. Moreover, patients who read the PIs have been found to be more likely to report adverse drug reactions (ADRs) [12, 13]. Higher levels of satisfaction among healthcare providers and adherence to the prescribed medicines by the patients have also been observed among those who receive their medicines accompanied by package inserts [7]. Dosage, ADRs, contraindications and shelf-life of the medications have been reported to be among the areas of focus upon reading the package inserts [10].

Despite the obvious usefulness of PIs, reports are available where PIs enclosed in commercial packs of medicinal products are not read as intended because of among other things, difficulty in understanding, presence of excessive not easy to understand information and text that is not legible [9]. Moreover, it has been indicated that the based on the language, font size, line spacing, length and complex lay-outs used, the PILs have been reported not to be user friendly to users especially the elderly and those with low literacy skills[14]. Absence of important information such as unclear dosage instructions, interactions, storage conditions, measures to be taken in case of overdose, inappropriate presentation of side effects and measures in case of side effects within the package inserts of medications have also been reported [15–17].

Studies have reported the need for clearer guidelines on writing of PILs, more details should be stipulated in order avoid the current challenges and variability in PILs from different manufacturers. These should include guidelines on recommendable font sizes, line spacing and offer more flexibility due to differences in medicines and contexts [14].

The shift from highly technical documents to patient-oriented package inserts is highly advocated towards optimizing the information for easy understanding among patients/clients [18]. Moreover, testing of optimized package inserts to the selected group of patients/clients before approval is increasingly advocated in jurisdictions of several countries with stringent systems for regulation of medicines [15]. The pre-testing by users has been observed to result into PILs which were perceived to be clearer and easy to use, thus indicating the essence of patient inputs during PILs development [14].

In East African community (EAC), the Partner States embarked on efforts to harmonize technical requirements for regulation of medicines in 2009 with ultimate publication of harmonized medicines regulation guidelines in November 2014 [19,20]. The guidelines also include a series of medicines registration provisions on quality, safety, efficacy and product information [21]. The guidelines have been successfully adopted in Kenya, Uganda and Tanzania which are the Partner States with functional National Medicines Regulatory Agencies (NMRAs), with the guidelines for format and content summary of product characteristics (SmPC), product labelling and patient information leaflets forming an important component of the medicines registration compendium. Despite the requirements, some variations in the composition and adequacy of information present on the package inserts have been observed in different products across the region. Discrepancies and lack of and/or availability of adequate information on package inserts medicinal products available on the markets of EAC Partner States has not been previously documented, this calls for need for a systematic research into this area guided or benchmarked on the existing regulatory frameworks.

The present study aimed at evaluating the level of conformity of manufacturers from within and outside of the EAC with harmonized product information requirements of the EAC Partner States. The study further aimed at evaluating similarities and differences within the Partner States’ NMRAs included in the study with respect to regulatory oversight in ensuring that relevant medicines information is included in medicinal products circulating on the respective markets.

## Methodology

### Selection of study medicines

Six tracer essential medicines (medicines selected to enable adequate representation of the phenomenon under observation) were selected for use in the determination of the extent to which manufacturers of generics and corresponding branded medicines do conform to package inserts requirements outlined in the EAC harmonized guidelines for registration of medicines [21]. The medicines were selected based on their indications as first line treatments, high rates of utilization within the medicines supply system and their positions in treatment of diseases of public importance across EAC Partner States [22,23].

Paracetamol was selected for being the most commonly used pain relief medication; Albendazole for its common use as an anthelminthic medicine. Artemether/Lumefantrine (ALu) was included in the study for being the first line medicine in the management and treatment of uncomplicated *Plasmodium falciparum* malaria. On the other hand, Ciprofloxacin and Amoxicillin were selected due to their pivotal role in the treatment of most community acquired bacterial infections including typhoid, urinary and respiratory tract infections among others. Moreover, each of the selected medicines are listed in the National Essential Medicine Lists of the countries included in this study [24–27].

### Sampling of PIs

Purposeful sampling was conducted, whereby medicines studied were selected on the basis outlined in section 2.1 above. A total of five to eight different generics commercial packs from each study medicine were purchased from the domestic markets in Kenya, Tanzania and Uganda. For each medicine, the corresponding branded product was also collected, and these included: Panadol^®^ (Paracetamol) from GlaxoSmithKline; Ciproxin^®^ (Ciprofloxacin) from Bayer; Coartem^®^(ALu) from Novartis; Amoxil^®^ (Amoxicillin) from GlaxoSmithKline; Flagyl^®^ (Metronidazole) from GD Searle LLC and Zentel^®^ (Albendazole) from GlaxoSmithKline. From collected packs of the products, a total of six branded product’s and ninety three generic product’s Product Information Leaflets were collected and assessed.

### Data collection

A special data collection tool was prepared to gather the information regarding presence of information on sixteen selected data sets mapping the key contents/requirements of PIs that were common in summary of product characteristics (SmPCs) of all branded products included in the study. The tool was used to capture information on *description and composition*, *indications*, *dosage and method of administration*, *contraindications*, *warning and precautions*, *side effects and adverse drug reactions*, *over-dosage*, *drug interactions*, *clinical pharmacology*, *use during pregnancy and lactation*, *excipients*, *storage conditions*, *shelf-life*, *container package description as well as instructions on handling and disposal of the medications*. The selected parameters are also part of the requirements of the harmonized EAC guidelines [21]

Package inserts were evaluated based on whether the parameters under observation were included or not included in the respective package insert. Moreover, the products were evaluated for their country of manufacturing and the market from which they were obtained.

### Data analysis

Data collected were analyzed using the Statistical Package for Social Sciences (SPSS) software, version 20. Proportions of the package inserts conforming to the selected parameters under observation were determined and compared. The comparisons aimed at determining the significance in differences in conformance of the branded and generic products, market from which the product was collected, region of origin of the manufacturer as well as between types of product. The differences were considered to be significant at P<0.05 using t-test on comparison of proportions.

## Results

### Details of the collected package inserts

A total of ninety-nine package inserts were obtained from packs of the samples collected from the markets in the three EAC Partner States. Of the selected six products, a corresponding branded product was also included in the study. Among the generic products; 41.9% (39/93), 36.6% (34/93) and 21.5% (20/93) PIs were collected from the medicines obtained in Uganda, Tanzania and Kenya, respectively.

**Fig 1:**
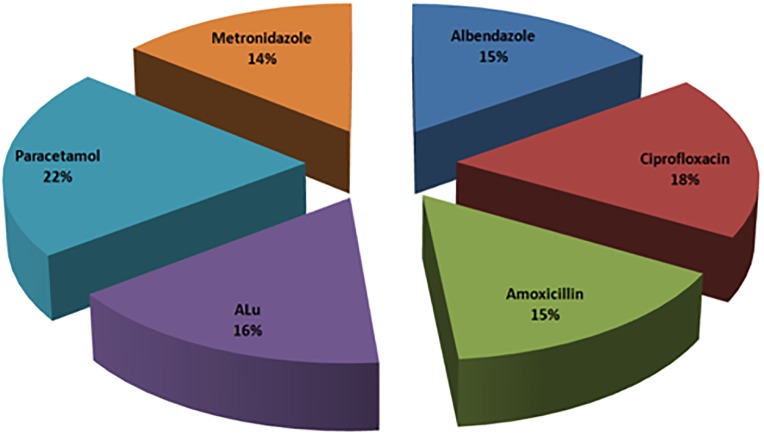
The percentage distribution of studied medicines based on their pharmacological group.

Based on the type of medicine almost reasonably equal numbers of PIs were collected for each study medicine as illustrated in Fig 1 below:

### General extent of conformity of all products to selected parameters

Overall, majority of the PIs did show high rate of conformity with regards to inclusion of the information on description and composition, indications, dosage and methods of administration, warnings and precautions, contra-indications and storage conditions, percentage conformity ranged from 93.9–100%.

**Fig 2:**
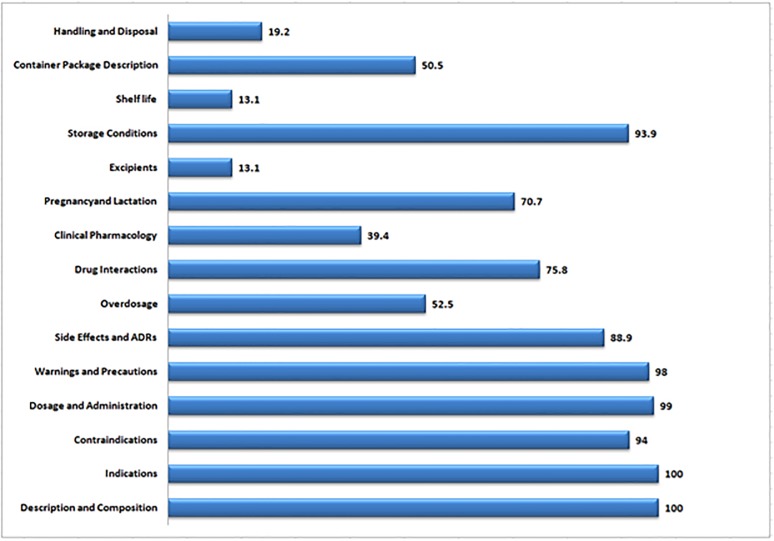
Overall percentage conformity of all collected medicines’ PIs to selected parameters.

However, the information on handling and disposal, container package description, excipients used, clinical pharmacology of the medicines, and directions regarding over dosage ranked the least in conformance with percentage conformity ranging from 13.1–52.5% (Fig 2).

### Conformity of generic products versus branded products in selected parameters

The percentage conformity of all the branded products was observed to be as high as 50–100% across all parameters (Table 1). However, generic products showed conformity of between 10.8–50.5% among five (5) of the parameters under evaluation. Moreover, percentage conformity of the branded products was found to be significantly higher (P<0.05) in the parameters of excipients, shelf-life as well as handling and disposal of medicines. S1 Table

**Table 1:** Percentage conformity of branded and generic medicines’ PIs to the parameters under assessment.

	PIs percentage conformity (%)		P values
Parameter	Branded (n = 6)	Generic (n = 93)	
Description and Composition	100	100	-
Indications	100	100	-
Contraindications	100	93.5	0.5214
Dosage and Administration	100	98.9	0.7972
Warnings and Precautions	100	97.8	0.7149
Side Effects and ADRs	100	88.2	0.3746
Overdose	83.3	50.5	0.1208
Drug Interactions	100	74.2	0.1550
Clinical Pharmacology	66.7	39.8	0.1971
Pregnancy and Lactation	100	68.8	0.1054
Excipients	50*	10.8*	0.0062
Storage Conditions	100	93.5	0.5214
Shelf life	50*	10.8*	0.0062
Container package Description	50	50.5	0.9812
Handling and Disposal	50*	17.2*	0.0491

*Proportions showing statistically significant differences at P< 0.05.

### Comparison of conformance to PI requirements by EAC Partner States

The percentage conformity of the products collected from each market did not show significant differences (P<0.05) from the overall proportion under each studied parameter. The parameters with high percentage of non-conformity in one EAC Partner State were observed to behave in a relatively similar way across Partner States (Table 2).

**Table 2:** Percentage conformity of products collected in three markets of the EAC Partner States.

	Market		
Parameter	Tanzania (n = 34)	Kenya (n = 20)	Uganda (n = 39)
Description and Composition	100	100	100
Indications	100	100	100
Contraindications	94.1	90	100
Dosage and Administration	97.1	100	94.9
Warnings and Precautions	100	100	100
Side Effects and ADRs	94.1	95	94.9
Overdose	52.9	35	56.4
Drug Interactions	73.5	75	74.4
Clinical Pharmacology	35.3	50	38.5
Pregnancy and Lactation	76.5	55	69.2
Excipients	8.8	10	18.8
Storage Conditions	97	100	87.2
Shelf life	11.8	0	15.4
Container Package Description	50	60	46.2
Handling and Disposal	14.7	15	20.5

### Conformance by country of manufacture of the products

Comparison of the percentage conformities of the package inserts of all studied products based on country of origin shows that PIs of the products manufactured in European countries have a high percentage of conformity in most of the parameters evaluated (Table 3). S2 Table

**Table 3:** Percentage non-conformity of manufactures in Tanzania, Uganda and Kenya.

	Manufacturer origin			
Parameters assessed	EAC (n = 20)	Asia (n = 63)	Europe (n = 16)	Combined (n = 99)
Description and Composition	100.0	93.7	100.0	100
Indications	100.0	98.4	100.0	100
Contraindications	65.0^#^	74.6^#^	100.0	94
Dosage and Administration	95.0	93.7	100.0	99
Warnings and Precautions	80.0	90.5	100.0	98
Side Effects and ADRs	85.0	84.1	100.0	88.9
Over dosage	15.0^#^	47.6	93.8^§^	52.5
Drug Interaction Drug interactions	45.0^#^	73.0	93.8	75.8
Clinical Pharmacology	35.0	36.5	56.3	39.4
Pregnancy and Lactation	35.0^#^	66.7	100.0	70.7
Excipients	0.0	4.8	56.3^§^	13.1
Storage Conditions	100.0	88.9	100.0	93.9
Shelf life	5.0	12.7	25.0	13.1
Container Package Description	55.0	42.9	43.8	50.5
Handling and Disposal	0.0^#^	15.9	37.5	19.2

^§^Statistically significantly higher percentage conformity compared to average of all PIs combined *(α = 0*.*05*, *n = 99)*.

^#^Statistically significantly lower percentage conformity compared to average of all PIs combined *(α = 0*.*05*, *n = 99)*.

### Overall conformity

The evaluation of the conformity of the products to the selected parameters indicated that Albendazole, Ciprofloxacin, Amoxicillin and ALu had generally above 50% conformity to product information requirements where the respective products complied to 11 out of 15 parameters assessed (Table 4) S5 Table.

**Table 4:** Percentage conformity of the selected study medicines as per their pharmacological groups obtained from the markets within the EAC Partner States.

	API						
Parameter	Albendazole (n = 15)	Ciprofloxacin (n = 18)	Amoxicillin (n = 15)	ALu (n = 16)	Paracetamol (n = 21)	Metronidazole (n = 14)	Combined (n = 99)
Description and Composition	100.0	100.0	100.0	100.0	100.0	100.0	100
Indications	100.0	100.0	100.0	100.0	100.0	100.0	100
Contraindications	93.3	100.0	93.3	100.0	81.0	100.0	94
Dosage and Administration	100.0	94.5	100.0	100.0	100.0	100.0	99
Warnings and Precautions	93.3	100.0	100.0	100.0	95.3	100.0	98
Side Effects and ADRs	93.3	96.1	93.3	100.0	90.5	100.0	88.9
Overdosage	53.3	77.8^§^	60.0	75.0	42.9	0.0	52.5
Drug Interactions	60.0	94.5	73.3	100.0^§^	66.7	57.1	75.8
Clinical Pharmacology	40.0	5.6^#^	73.3^§^	87.5^§^	42.9	0.0^#^	39.4
Pregnancy and Lactation	86.7	88.9	80.0	100.0^§^	19.0^#^	64.3	70.7
Excipients	20.0	27.8	0.0	0.0	19.1	7.1	13.1
Storage Conditions	86.7	88.9	93.3	100.0	100.0	92.9	93.9
Shelf life	46.7A	0.0	6.7	0.0	23.8	0.0	13.1
Container Package Description	86.7A	50.0	13.3a	0.0^#^	71.4	78.6^§^	50.5
Handling and Disposal	40.0	0.0^#^	46.7^§^	37.5	0.0^#^	0.0	19.2

^§^Statistically significantly higher percentage conformity compared to average of all PIs combined *(α = 0*.*05*, *n = 99)*.

^#^Statistically significantly lower percentage conformity compared to average of all PIs combined *(α = 0*.*05*, *n = 99)*.

## Discussion

The findings indicated lack of proper instructions on handling and disposal of medications. This may lead to poisoning to incidences as well as increasing chances of developing resistant microbes due to exposure to sub-optimal doses [27].

The study revealed only 13.1% (13/99) conformances in inclusion of shelf life and disclosure of excipients present in the formulation. These pose the risk for poisoning, adverse drug reactions, development of drug resistance, as well as treatment failure due to exposure to harmful degradation products, lower medicine’s content and unwanted excipients to some individuals.

The study further showed that only 39.4% (39/99) of the studied medicines included the section on clinical pharmacology in their PIs. Moreover, the information regarding actions to be taken in case of over dosage was found to be included in 52.5% (52/99) of the studied medicines. Absence of such information may result into irrational prescribing and dispensing and hence affect the overall treatment outcomes [10, 17] and bring about detrimental consequences to patients in events of over dosage [28].

Performance of the branded products in majority of the remaining parameters was generally found to be higher than those of their generic counterparts regardless of the lack of statistical significance. This is contributed by their prior assessment and approval in countries with stringent regulatory requirements for regulation of medicines [29–30]. The pattern observed in generic products may be attributed by outmost priority on indications, dosage and methods of administration given by generic manufacturers compared to other sections of the PI.

High conformity of 73.5–100% was seen in description and composition of the product, indications, contraindications, dosage and method of administration, warning and precautions, side effects and ADRs, drug interactions and storage conditions. Moreover, the consistent low percentage conformity was observed in parameters such as information on excipients, shelf-life as well as handling and disposal of unused medicines in each of the Partner State. The results suggest that regulators in the three Partner States have generally comparable level of scrutiny and approaches on product information requirements of medicines registered in their respective markets. The results are suggestive of the need for a collective improvement in the regulatory review and enforcement in individual Partner States and at regional level with respect to conformance to product information requirements.

Among the Europe based manufactures, the percentage conformity in overdose and excipients parameters was determined to be significantly (P<0.05) above the rate observed in the PIs enclosed in the medicines manufactured elsewhere. Moreover, the manufactures from Asia, mainly India and China, exhibited a relatively better conformity profile across parameters compared to those from EAC Partner States. While there was a statistically higher percentage conformity in contraindications parameter among Asian manufacturers (P<0.05) compared to manufacturers from EAC Partner states, significantly low conformities (P<0.05) in a total of four parameters: contraindications, over dosage, drug interaction, pregnancy and lactation as well as handling and disposal of unused medicines was observed in medicines from EAC based manufacturers compared to those manufactured in Asia and Europe. S3 Table

These findings suggest the presence of a notable relationship between the country of origin of the product and the extent of conformity to the inclusion of adequate amount of the essential information in the medicines’ package inserts. Inadequate conformance observed in PIs of products from EAC based manufacturers may be attributed by the fact that manufacturers from the respective part of the world have not put much emphasis on important aspects pertaining to proper handling and use of the product after batch release, and this may be attributed by the myths among the respective, that generic medicines are absolutely safe and inclusion of detailed information may not add value to the patients. In addition, the region has been reported to have low number of highly skilled regulatory professionals working in the industries who would otherwise advise on aspects of pharmaceutical business that is beyond batch manufacturing and release.

Moreover, ALu indicated a 100% conformity in 9 out of the 15 observed parameters, while Albendazole indicated none of its parameters to be significantly (P<0.05) lower in percentage conformity when compared to the other studied medicines See S1 and S4 Tables. These findings may be associated with the extensive use of ALu and Albendazole in vertical programmes for control of malaria and lymphatic phillariasis and helminthic eradication programmes, respectively across the Partner States. These Programmes are likely to influence conformity to a higher degree among manufacturers due to a closer follow up of their products by regulators, international procurement agencies, donors and respective ministries of health.

The findings have also indicated three parameters had lowest conformity across all the assessed medicines. These included excipients (0–27.8%), shelf life (0–46.7%) and handling and disposal (0–46.7%). This may indicate low emphasis on the respective aspects of product information requirements by regulators in the region. Significant low level of conformity regarding the inclusion of information on handling and disposal observed in PIs of Paracetamol, Ciprofloxacin and Metronidazole poses the chances of poor handling of the respective products. For the later two products, effects could include increased antimicrobial resistance and eventually negative consequences considering pivotal role of these medicines in treatment of many community acquired infections.

On the other hand, regardless of their high utilization patterns, none of the evaluated samples for Amoxicillin and ALu was found to contain information regarding the list of excipients included in the respective formulations. Even though recent Cohort Event Monitoring (CEM) study done in Tanzania revealed generally good tolerability and safety of ALu, few adverse drug reactions have been reported which may not all be attributed to the constituent drug substances, but rather certain excipients in the formulations (23). Moreover, no significant limitations were encountered during the study.

## Conclusion and recommendations

The findings of our study revealed existence of significant number of medicinal products circulating on the markets of EAC Partner States without necessarily complying with all product information requirements as agreed by these countries in the harmonized regional guidelines as well as individual Partner States guidelines. Branded medicines have demonstrated overall higher conformity to requirements compared to their generics counterparts. Generally consistent high degree of conformity in some parameters and low conformity in other parameters across all groups of the studied medicines was also observed.

The study has also revealed a general trend in the level of conformity with respect to country of manufacture of the medicines, where medicines manufactured in countries with stringent medicines regulatory systems (SRAs) have shown comparatively higher degree of conformity with product information requirements while those manufactured by EAC manufacturers were the least. Moreover, the study revealed absence of much needed critical product information in essential products with higher rate of consumption and that are needed for treatment of most common community-acquired infections in the EAC Partner States.

The study has revealed gross inadequacy of important product information in the commercial packs of medicines manufactured in the EAC Partner States and distributed in the respective countries. While there is a will by the national governments and regulators of the respective countries to promote domestic manufacturing by allowing the respective manufacturers to operate at minimum levels of standards, some of the conformance to product information requirements does not require significant financial investment to implement. Since all aspects of medicines information are critical for proper prescribing, dispensing, storage and use of the products, emphasis on having adequate and thorough information on all medicines given to the patients is of paramount importance for protection of health of the patients and realizing maximum benefits of treatment regimen, and hence compulsory inclusion of detailed and accurate information should be emphasized.

We therefore recommend that national medicine regulatory agencies (NMRAs) should take deliberate efforts to ensure that thorough pre-market assessment of product information to be accompanied in commercial packs of medicinal products is conducted at the time of registration. In addition, the NMRAs are advised to strengthen their post marketing surveillance programmes to increase frequencies and coverage in order to ensure that medicines circulating on the markets of the respective countries comply to among other things, medicine information requirements at all times.

Moreover, emphasis should be given to manufacturers on the importance of inclusion of appropriate and adequate product information for the safety of patients. This include advocating for inclusion of patient friendly and easy to understand medicines information for those medicinal products. The study also recommends further research on the adequacy of current medicines information requirements and explore on the mechanisms through which the information translates in medicines use patterns among health care practitioners and patients.

## Supporting information

S1 Table Innovator and generic products from 3 different markets vs conformity to selected parameters. (PDF)

S2 Table Region of the manufacturer vs conformity to selected parameters. (PDF)

S3 Table Market collected (all products combined) vs parameters. (PDF)

S4 Table Innovator and generic (all APIs combined) vs selected parameters. (PDF)

S5 Table Individual APIs vs conformity to selected parameters. (PDF)

## References

[1] Shivkar YM. Clinical information in drug package inserts in India. Journal of postgraduate medicine. 2009 4 1;55(2):104 doi: 10.4103/0022-3859.52840 19550054 10.4103/0022-3859.52840

[2] Bhosale UA. Evaluation of Knowledge and Awareness of Patients about Prescribed Drugs and their Package Inserts: A Cross-sectional Study. Asian Journal of Pharmaceutics (AJP): Free full text articles from Asian J Pharm. 2016 6 27;10(2).

[3] Young A, Tordoff J, Smith A. ‘What do patients want?’Tailoring medicines information to meet patients’ needs. Research in Social and Administrative Pharmacy. 2017 11 1;13(6):1186–90. doi: 10.1016/j.sapharm.2016.10.006 27818214 10.1016/j.sapharm.2016.10.006

[4] Joubert P, Lasagna L. Patient package inserts. I. Nature, notions, and needs. Clinical Pharmacology & Therapeutics. 1975 11 1;18(5part1):507–13. 1183137 10.1002/cpt1975185part1507

[5] Vandierendonck A, De Vooght G, Reynvoet B, Lammertyn J, Vander Stichele RH. Impact of benefit messages in patient package inserts on subjective drug perception. Drug information journal. 2002 1;36(1):201–8

[6] Joubert P, Lasagna L. Patient package inserts. II. Toward a rational patient package insert. Clinical Pharmacology & Therapeutics. 1975 12 1;18(6):663–9. 812628 10.1002/cpt1975186663

[7] Gibbs SH, Waters WE, George CF. The benefits of prescription information leaflets (2). British journal of clinical pharmacology. 1989 9 1;28(3):345–51. 2571354 10.1111/j.1365-2125.1989.tb05436.x PMC1379954

[8] Zaid AN, Sweileh WM. Attitudes of consumers and healthcare professionals towards the patient package inserts-a study in Palestine. Pharmacy practice. 2012 3 12;10(1):57–63. 24155818 10.4321/s1886-36552012000100010 PMC3798163

[9] Bandesha G, Raynor DK, Teale C. Preliminary investigation of patient information leaflets as package inserts. International Journal of Pharmacy Practice. 1996 12 1;4(4):246–8.

[10] Vander Stichele RH, Van Haecht CH, Braem MD, Bogaert MG. Attitude of the public toward technical package inserts for medication information in Belgium. DICP. 1991 9;25(9):1002–6.). 1949957 10.1177/106002809102500916

[11] Hamrosi KK, Aslani P, Raynor DK. Beyond needs and expectations: identifying the barriers and facilitators to written medicine information provision and use in Australia. Health Expectations. 2014 4 1;17(2):220–31 doi: 10.1111/j.1369-7625.2011.00753.x 22390211 10.1111/j.1369-7625.2011.00753.x PMC5060720

[12] Vander Stichele R, De Backer G, Bogaert MG. Impact of patient package inserts on patients’ satisfaction, adverse drug reactions and risk perception: the case of NSAIDs for posttraumatic pain relief. Patient Education and Counseling. 1991 6 1;17(3):205–15.

[13] Haecht CH, Vander Stichele R, Bogaert MG. Package inserts for antihypertensive drugs: use by the patients and impact on adverse drug reactions. European journal of clinical pharmacology. 1990 12 1;39(6):551–4. 2095340 10.1007/BF00316093

[14] van Dijk L, Monteiro SP, Vervloet M, de Bie J, Raynor DT. Study on the package leaflets and the summaries of product characteristics of medicinal products for human use. PIL’s Study. European Union Google Scholar. 2014 Jul

[15] Fuchs J, Hippius M, Schaefer M. Analysis of German package inserts. International journal of clinical pharmacology and therapeutics. 2006 1 1;44(1):8 16425965 10.5414/cpp44008

[16] Al-aqeel SA. Evaluation of medication package inserts in Saudi Arabia. Drug, healthcare and patient safety. 2012;4:33 doi: 10.2147/DHPS.S29402 22570572 10.2147/DHPS.S29402 PMC3345877

[17] Hermann F, Herxheimer A, Lionel ND. Package inserts for prescribed medicines: what minimum information do patients need?. Br Med J. 1978 10 21;2(6145):1132–5. 709267 10.1136/bmj.2.6145.1132 PMC1608236

[18] Vander Stichele RH, De Potter B, Vyncke P, Bogaert MG. Attitude of physicians toward patient package inserts for medication information in Belgium. Patient education and counseling. 1996 6 1;28(1):5–13. 8852202 10.1016/0738-3991(96)00866-x

[19] FM‘t Hoen E, Hogerzeil HV, Quick JD, Sillo HB. A quiet revolution in global public health: The World Health Organization’s Prequalification of Medicines Programme. Journal of public health policy. 2014 5 1;35(2):137–61. doi: 10.1057/jphp.2013.53 24430804 10.1057/jphp.2013.53

[20] Ndomondo-Sigonda M, Ambali A. The African medicines regulatory harmonization initiative: rationale and benefits. Clinical Pharmacology & Therapeutics. 2011 2 1;89(2):176–8. 21252936 10.1038/clpt.2010.299

[21] The Compendium of medicine evaluation and registration for medicine regulation harmonization in the East African community, 2014.

[22] Hotez PJ, Kamath A. Neglected tropical diseases in sub-saharan Africa: review of their prevalence, distribution, and disease burden. PLoSNegl Trop Dis. 2009 8 25;3(8):e412 10.1371/journal.pntd.0000412 PMC2727001 19707588

[23] Mssusa AK, Fimbo AM, Nkayamba AF, Irunde HF, Sillo HB, Shewiyo DH et al Safety Profile of Artemether-Lumefantrine: A Cohort Event Monitoring Study in Public Health Facilities in Tanzania. Clin Drug Investig. 2016 5; 36(5); 401–11 doi: 10.1007/s40261-016-0385-z 26951203 10.1007/s40261-016-0385-z

[24] The United Republic of Tanzania, Ministry of Health and Social Welfare. Standard Treatment Guidelines and Essential Medicines List, Fourth Edition 5, 2013

[25] Ministry of Health, Kenya Essential Medicines List 2016, June 2016

[26] Ministry of Health, Republic of Uganda, Essential Medicines and Health Supplies List for Uganda (EMHSLU), 2012

[27] Popowska M, Miernik A, Rzeczycka M, Łopaciuk A. The impact of environmental contamination with antibiotics on levels of resistance in soil bacteria. J Environ Qual. 2010 Sep-Oct;39 (5):1679–87. 21043273 10.2134/jeq2009.0499

[28] Centres for Disease Control and Prevention. CDC Grand Rounds: Prescription Drug Overdoses—a U.S. Epidemic. Weekly. January 13, 2012 / 61(01);10–13. www.cdc.gov (accessed on 21st March 2017) 22237030

[29] Drugs@FDA: FDA Approved Drug Products. http://www.accessdata.fda.gov/scripts/cder/daf/ (accessed 21/03/2017)

[30] European Medicines Agency. (EMA) Approved medicines. http://www.ema.europa.eu/ema/index.jsp?curl=pages/includes/medicines/medicines_landing_page.jsp (accessed 21/03/2017)

