# Supplementary material for: Conformity of package inserts information to regulatory requirements among selected branded and generic medicinal products circulating on the East African market
Source: PLoS One. 2018 May 22;13(5):e0197490. doi: 10.1371/journal.pone.0197490 (PMC5963798; doi:10.1371/journal.pone.0197490)
Supplement: S1 Table — (PDF) [file pone.0197490.s001.pdf]

		Status				
		Innovator			Generic	
		Market Collected			Market Collected	
		Tanzania	Kenya	Uganda	Tanzania	Kenya
		Count	Count	Count	Count	Count
	NOT MET	0	0	0	0	0
	PARTIALLY MET	0	0	0	1	1
	MET	6	0	0	33	19
Indications	NOT MET	0	0	0	0	0
	PARTIALLY MET	0	0	0	1	0
	MET	6	0	0	33	20
Contraindications	NOT MET	0	0	0	2	2
	1	0	0	0	1	0
	PARTIALLY MET	0	0	0	6	2
	MET	6	0	0	25	16
	NOT MET	0	0	0	1	0
	PARTIALLY MET	0	0	0	2	1
	MET	6	0	0	31	19
	NOT MET	0	0	0	0	0
	PARTIALLY MET	0	0	0	5	2
	MET	6	0	0	29	18

Side Effects and ADRs	NOT MET	0	0	0	2	1
	PARTIALLY MET	0	0	0	1	0
	MET	6	0	0	31	19
Overdosage	NOT MET	1	0	0	16	13
	PARTIALLY MET	0	0	0	1	1
	MET	5	0	0	17	6
Drug Interactions	NOT MET	0	0	0	9	5
	PARTIALLY MET	0	0	0	3	1
	MET	6	0	0	22	14
Clinical Pharmacology	NOT MET	2	0	0	22	10
	PARTIALLY MET	0	0	0	1	1
	MET	4	0	0	11	9
Pregnancyand Lactation	NOT MET	0	0	0	8	9
	PARTIALLY MET	0	0	0	3	1
	MET	6	0	0	23	10
