# Supplementary material for: Conformity of package inserts information to regulatory requirements among selected branded and generic medicinal products circulating on the East African market
Source: PLoS One. 2018 May 22;13(5):e0197490. doi: 10.1371/journal.pone.0197490 (PMC5963798; doi:10.1371/journal.pone.0197490)
Supplement: S2 Table — (PDF) [file pone.0197490.s002.pdf]

		Region of Manufacuturer	
		EAC	Abroad
		Count	Count
	NOT MET	0	0
	PARTIALLY MET	0	4
	MET	16	79
Indications	NOT MET	0	0
	PARTIALLY MET	0	1
	MET	16	82
Contraindications	NOT MET	4	2
	1	0	1
	PARTIALLY MET	1	15
	MET	11	65
	NOT MET	0	1
	PARTIALLY MET	0	4
	MET	16	78
	NOT MET	0	2
	PARTIALLY MET	3	5
	MET	13	76
Side Effects and ADRs	NOT MET	2	9
	PARTIALLY MET	0	2
	MET	14	72

Overdosage	NOT MET	13	34
	PARTIALLY MET	0	4
	MET	3	45
Drug Interactions	NOT MET	9	15
	PARTIALLY MET	0	5
	MET	7	63
Clinical Pharmacology	NOT MET	10	48
	PARTIALLY MET	2	0
	MET	4	35
Pregnancy and Lactation	NOT MET	9	20
	PARTIALLY MET	0	5
	MET	7	58
	NOT MET	14	72
	PARTIALLY MET	0	0
	MET	2	11
Excipients	NOT MET	16	70
	PARTIALLY MET	0	1
	MET	0	12
