# Supplementary material for: Conformity of package inserts information to regulatory requirements among selected branded and generic medicinal products circulating on the East African market
Source: PLoS One. 2018 May 22;13(5):e0197490. doi: 10.1371/journal.pone.0197490 (PMC5963798; doi:10.1371/journal.pone.0197490)
Supplement: S3 Table — (PDF) [file pone.0197490.s003.pdf]

		Market Collected		
		Tanzania	Kenya	Uganda
		Count	Count	Count
	NOT MET	0	0	0
	PARTIALLY MET	1	1	2
	MET	39	19	37
Indications	NOT MET	0	0	0
	PARTIALLY MET	1	0	0
	MET	39	20	39
Contraindications	NOT MET	2	2	2
	1	1	0	0
	PARTIALLY MET	6	2	8
	MET	31	16	29
	NOT MET	1	0	0
	PARTIALLY MET	2	1	1
	MET	37	19	38
	NOT MET	0	0	2
	PARTIALLY MET	5	2	1
	MET	35	18	36
Side Effects and ADRs	NOT MET	2	1	8
	PARTIALLY MET	1	0	1
	MET	37	19	30
