# Supplementary material for: Conformity of package inserts information to regulatory requirements among selected branded and generic medicinal products circulating on the East African market
Source: PLoS One. 2018 May 22;13(5):e0197490. doi: 10.1371/journal.pone.0197490 (PMC5963798; doi:10.1371/journal.pone.0197490)
Supplement: S5 Table — (PDF) [file pone.0197490.s005.pdf]

		API			
		Albendazole	Ciprofloxacin	Amoxicillin	ALu
		Count	Count	Count	Count
	NOT MET	0	0	0	0
	PARTIALLY MET	0	1	0	3
	MET	15	17	15	13
Indications	NOT MET	0	0	0	0
	PARTIALLY MET	0	0	1	0
	MET	15	18	14	16
Contraindications	NOT MET	1	0	1	0
	1	0	0	0	1
	PARTIALLY MET	2	3	1	6
	MET	12	15	13	9
	NOT MET	0	1	0	0
	PARTIALLY MET	0	1	2	0
	MET	15	16	13	16
	NOT MET	1	0	0	0
	PARTIALLY MET	0	0	1	4
	MET	14	18	14	12
Side Effects and ADRs	NOT MET	1	7	1	0
	PARTIALLY MET	2	0	0	0
	MET	12	11	14	16
